# No causal association between pneumoconiosis and three inflammatory immune diseases: a Mendelian randomization study

**DOI:** 10.3389/fpubh.2024.1373044

**Published:** 2024-03-27

**Authors:** Yu-Jie Du, Zhang-Wei Lu, Kai-Di Li, Yi-Yu Wang, Hong Wu, Rong-Gui Huang, Xue Jin, Yi-Yuan Wang, Jing Wang, An-Yi Geng, Bao-Zhu Li

**Affiliations:** ^1^Department of Epidemiology and Biostatistics, School of Public Health, Anhui Medical University, Hefei, China; ^2^Anhui Provincial Laboratory of Inflammatory and Immune Diseases, Hefei, China; ^3^Second Affiliated Hospital of Anhui Medical University, Hefei, China

**Keywords:** inflammatory immune diseases, causal relationship, rheumatoid arthritis, systemic lupus erythematosus, gout

## Abstract

**Objectives:**

To investigate the causal relationships between pneumoconiosis and rheumatoid arthritis (RA), systemic lupus erythematosus (SLE), and gout.

**Methods:**

The random-effects inverse variance weighted (IVW) approach was utilized to explore the causal effects of the instrumental variables (IVs). Sensitivity analyses using the MR-Egger and weighted median (WM) methods were did to investigate horizontal pleiotropy. A leave-one-out analysis was used to avoid the bias resulting from single-nucleotide polymorphisms (SNPs).

**Results:**

There was no causal association between pneumoconiosis and SLE, RA or gout in the European population [OR = 1.01, 95% CI: 0.94–1.10, *p* = 0.74; OR = 1.00, 95% CI: 0.999–1.000, *p* = 0.50; OR = 1.00, 95% CI: 1.000–1.001, *p* = 0.55]. Causal relationships were also not found in pneumoconiosis due to asbestos and other mineral fibers and SLE, RA and gout [OR = 1.01, 95% CI: 0.96–1.07, *p* = 0.66; OR = 1.00, 95% CI: 1.00–1.00, *p* = 0.68; OR = 1.00, 95% CI: 1.00–1.00, *p* = 0.20].

**Conclusion:**

Our study suggests that pneumoconiosis may have no causal relationship with the three inflammatory immune diseases.

## 1 Introduction

The inflammatory immune response is a vital immune defense mechanism. Tissues go through a normal process involving self-defense and damage repair when they are damaged, or infected by toxins or bacteria, or caused by other factors (1). An excessive inflammatory immune response will cause body damage, leading to inflammatory immune diseases, including rheumatoid arthritis (RA), systemic lupus erythematosus (SLE), gout, and so on. It has been a significant public health issue that has had a negative impact on both long-term social and economic development as well as human health. This class of disorders is characterized by systemic inflammatory immunological dysfunction, but the precise cause and mechanism are yet unknown. It has been revealed that occupational exposure to particulate matter and environmental variables are significant contributors to the pathogenesis of this disease.

Occupational populations might develop inflammatory immunological disorders. People exposed to silica, for instance, have a relative risk of RA that is more than three times higher (2). Of those occupational populations with RA, SLE, and other disorders, 14.1% are extensively exposed to dust, chemicals, and other inorganic and organic pollutants. Immune disorders were substantially correlated with overall occupational environmental exposure [OR (95%CI) = 1.29 (1.11–1.49)] (3). Exposure to inorganic dust, such as silica and asbestos, is the most likely cause of pneumoconiosis and further contributes to a relatively high incidence rate ratio of immune system diseases (1.99) (3). And numerous epidemiological studies have demonstrated that pneumoconiosis has a strong correlation with the development of gout, RA, SLE, and other inflammatory immunological disorders. A nationwide cohort study had indicated that miners exposed to silica have an increased possibility of developing SLE and RA (4). In Japan, reports regarding thirty cases of pneumoconiosis with RA, SLE, and other illnesses have been presented (5). Patients with RA who are exposed to inorganic dust combined with several distinct pulmonary nodules, mainly peripheral to the lung, is called Caplan’s syndrome (6). At the time of diagnosis, a large number of individuals with Caplan’s syndrome had at least mild pneumoconiosis (7). According to a case–control study conducted in Sweden, women who are exposed to dust have an increased probability of developing gout and may also be at risk for other inflammatory disorders (8). This might be relevant to the mechanism of hyperuricemia, the process of sodium urate crystallization, or the inflammatory reaction to the crystals after prolonged exposure to dust. Pneumoconiosis patients have been reported to have a higher level of polyclonal gamma-globulin, particularly IgG, and a high positivity rate for autoantibodies such anti-nuclear antibodies (9). Pneumoconiosis is associated with increased autoantibodies, immune complexes, and overproduction of immunoglobulins, including rheumatoid factors (10, 11). Additionally, research on animals has demonstrated that crystalline silica (cSiO_2_) increased the risk of immunological disorders (12). Following a week-long exposure to cSiO2, the mice had highly elevated TNF-α levels, acquired autoantigens associated with lupus, and decreased levels in blood IgG, a hallmark of systemic inflammation. And it triggered autoantibodies against the autoantigens—collagen II, fibronectin, etc.—that are linked to RA. Besides, acute exposure to cSiO2 in lupus-prone mice conduced to lung inflammation, the generation of pro-inflammatory cytokines, and the activation of B- and T-cells, all of which hasten the onset of autoimmune disorders (13).

Nevertheless, Studies have constraints in their ability to detect and investigate isolated exposures and are unable to confirm the pathophysiology or causation of pneumoconiosis and inflammatory immune disorders, despite indications of a link between the two diseases. Two-sample Mendelian randomization (MR) method, as an effective method using genetic variation as instrumental variables (IVs), has been used to explore the causal associations between inflammatory immune diseases and other diseases, such as healthy lifestyle, atopic dermatitis, and hormonal factors (14–16). Genetic variation could minimize confounding, give stronger causal reasoning, and overcome some of the limitations of classic observational research since it is unaffected by other variables or the external environment. Therefore, we explored into the causative connections between pneumoconiosis and gout, SLE, and RA using the MR analysis.

## 2 Methods

### 2.1 Study design and instrument selection

**Figure 1:**
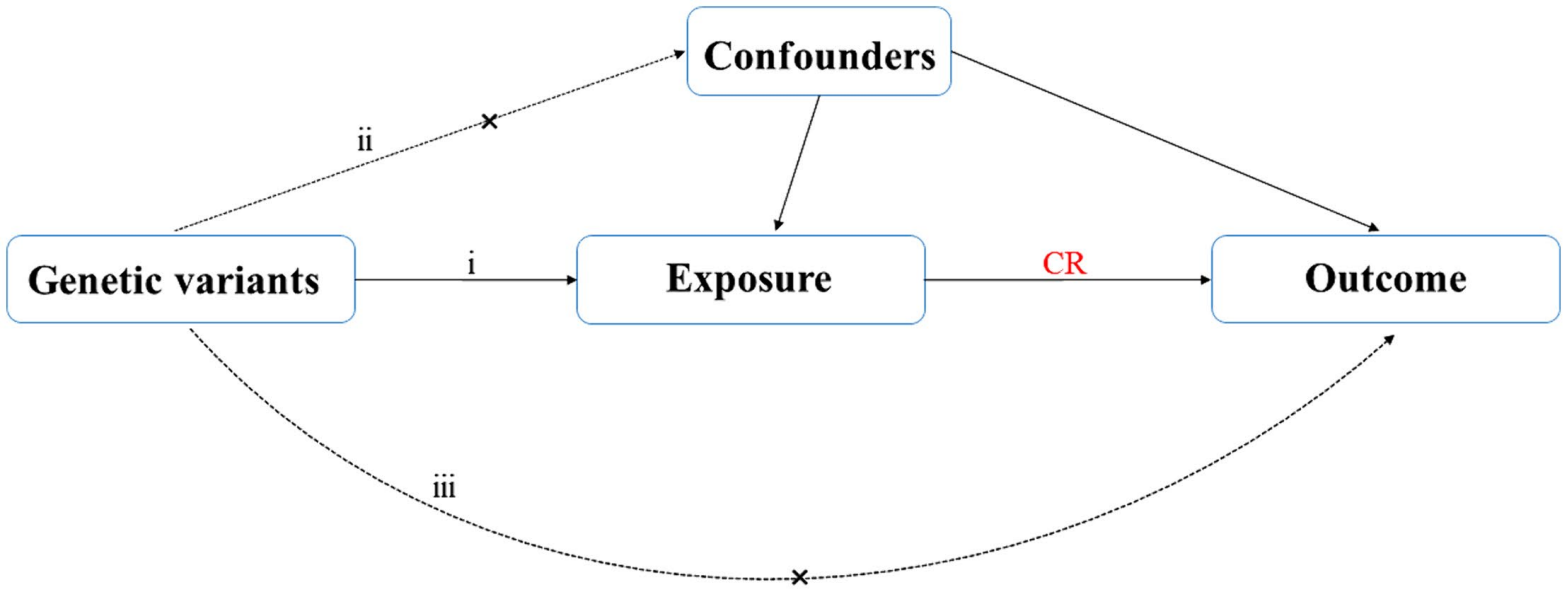
Diagram of MR study design.

**Figure 2:**
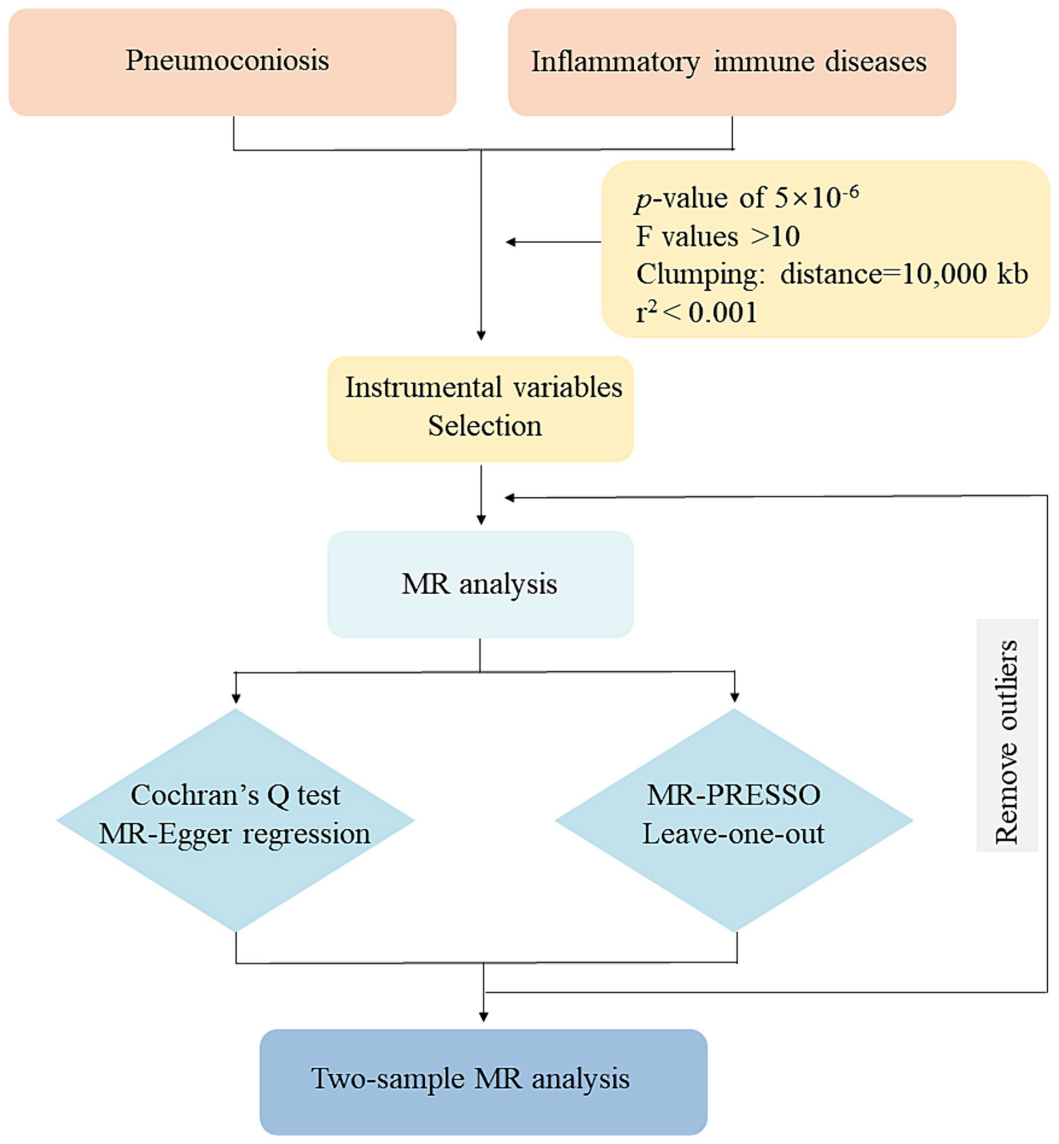
Flowchart of this MR study. MR: Mendelian randomization; MR-PRESSO: MR-pleiotropy residual sum and outlier.

A two-sample MR study was conducted to find causal relationships. The two-sample MR package in R (version 4.2.3) was used. Since our analysis was based on aggregated summary-level data, ethical approval was unneeded. Figure 1 illustrates our study’s assumptions and design. The MR analysis had its basis on three primary assumptions: (1) IVs are supposed to be strongly linked with exposures; (2) they should be independent from confounding factors associated with the relationship between the exposures and outcomes; (3) relevance only through exposures to influence the results (17). The flowchart of our study design is shown in Figure 2.

Regarding IVs, we chose SNPs that showed a substantial exposure association (*p* < 5 × 10^−6^). And the *F* and *r*^2^ values were calculated to ensure each SNP’s validity. Linkage disequilibrium (LD) was eliminated in our study (*r*^2^ < 0.001, 10,000 kb). Palindromic SNPs with intermediate allele frequencies and SNPs with *F*-values <10 were removed (18). A comprehensive summary of the SNPs that were substantially linked to our exposures was presented in Supplementary Tables S1–S3.

### 2.2 Data sources

All GWAS data was obtained from the IEU.^1^ Two exposures fall into one occupational disease and the GWAS data was from the FinnGen consortium^2^ and the European Bioinformatics Institute GWAS Catalogue.^3^ The exposures we selected including pneumoconiosis (ID: ebi-a-GCST90018900) and pneumoconiosis due to asbestos and other mineral fibers (ID: finn-b-J10_ASBESTPNEUMOC).

Three outcomes’ GWAS data were all from the European Bioinformatics Institute GWAS Catalogue. The inflammatory immune diseases considered to be analyzed were as follows: SLE (ID: ebi-a-GCST90018917), RA (ID: ebi-a-GCST90038685), and gout (ID: ebi-a-GCST90038687). Since all of the samples were European in origin, there were no racial differences. Table 1 presents the exposure and outcome summary information.

**Table 1:** Descriptive details and Mendelian randomization estimates of pneumoconiosis and SLE, RA and gout.

Outcome	Exposure	Ancestry	Methods	OR	*p*-value	MR-Egger intercept (*p*-value)	Cases		Controls		Sample size		Sources	
							Exposure	Outcome	Exposure	Outcome	Exposure	Outcome	Exposure	Outcome
SLE	Pneumoconiosis	European	IVW	1.01 (0.94–1.10)	0.74	0.05 (0.23)	433	647	478,607	482,264	479,040	482,911	EBI	EBI
			MR-Egger	0.93 (0.90–1.13)	0.37									
			WM	1.01 (0.80–1.08)	0.87									
	Pneumoconiosis due to asbestos and other mineral fibers		IVW	1.01 (0.96–1.07)	0.66	−0.01 (0.88)	235		216,866		217,101		FinnGen	
			MR-Egger	1.02 (0.92–1.13)	0.72									
			WM	0.99 (0.92–1.07)	0.80									
RA	Pneumoconiosis	European	IVW	1.00 (1.00–1.00)	0.50	0.00 (0.66)	433	5,427	478,607	479,171	479,040	484,598	EBI	EBI
			MR-Egger	1.00 (1.00–1.00)	0.48									
			WM	1.00 (1.00–1.00)	0.22									
	Pneumoconiosis due to asbestos and other mineral		IVW	1.00 (1.00–1.00)	0.68	0.00 (0.19)	235		216,866		217,101		FinnGen	
	Fibers		MR-Egger	1.00 (1.00–1.00)	0.43									
			WM	1.00 (1.00–1.00)	0.92									
Gout	Pneumoconiosis	European	IVW	1.00 (1.00–1.00)	0.50	0.00 (0.66)	433	6,810	478,607	477,788	479,040	484,598	EBI	EBI
			MR-Egger	1.00 (1.00–1.00)	0.48									
			WM	1.00 (1.00–1.00)	0.22									
	Pneumoconiosis due to asbestos and other mineral		IVW	1.00 (1.00–1.00)	0.68	0.00 (0.19)	235		216,866		217,101		FinnGen	
	fibers		MR-Egger	1.00 (1.00–1.00)	0.43									
			WM	1.00 (1.00–1.00)	0.92									

EBI: European Bioinformatics Institute; IVW: inverse variance weighted; WM: Weighted median; OR: odd ratios; CI: CONFIDENCE interval.

### 2.3 Statistical analysis

The MR-Egger method, weighted median (WM), inverse variance weighted (IVW) method, simple mode, and weighted mode were the methods employed. And the main analytical approach used to evaluate the causal links was the IVW method. Besides, the chosen SNPs had no connection to smoking or alcohol consumption, which may be confounding factors for inflammatory immune diseases.

For validation of the accuracy of the results we obtained, sensitivity analyses involving the Cochran’s Q test, MR-PRESSO, MR-Egger regression, and leave-one-out analysis were performed. Cochran’s Q test was utilized to assess SNPS heterogeneity (*p* > 0.05 indicates no heterogeneity) (19). Genetic diversity that influences outcomes through alternative pathways is known as pleiotropy (20). With MR-PRESSO, pleiotropy at the gene level was examined. It adjusts for the influence of genetic variants on causal estimates and identifies variants with pleiotropic effects. The presence of pleiotropy was also assessed by applying MR-Egger regression. An intercept that is statistically significant is able to identify pleiotropy. It computes the linear regression of the genetic variations on the outcome. To assess the overall influence of certain genetic variations, a leave-one-out analysis was used. The removal of one genetic variant at a time can be done with this method. Furthermore, by recalculating the causal estimate, the effect of each unique genetic variation may be determined (21).

## 3 Results

### 3.1 MR analysis of pneumoconiosis on SLE

**Figure 3:**
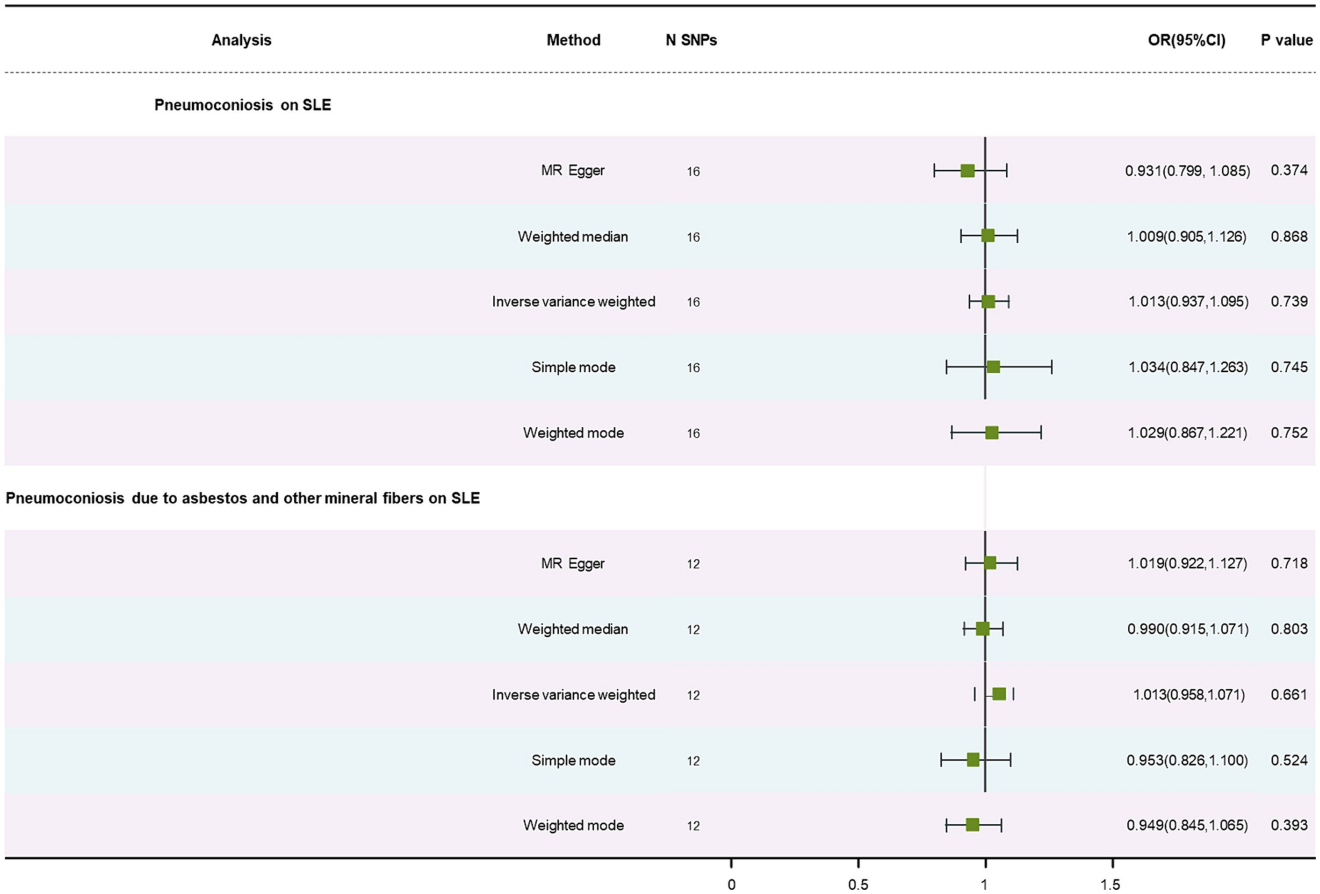
Forest plot of SNPs associated with pneumoconiosis and pneumoconiosis due to asbestos and other mineral fibers and their risk on SLE.

No causal association was discovered in results of pneumoconiosis, pneumoconiosis due to asbestos and other mineral fibers on SLE, listed in Table 1, using the IVW method (OR: 1.01, *p* = 0.74; OR: 1.01, *p* = 0.66). This correlation was also not detected using the WM method and MR-Egger approach (Supplementary Figures S1A,B). We consider that our results are credible because there was no significant heterogeneity (*p* = 0.50; *p* = 0.57) and no horizontal pleiotropy (*p* = 0.23; *p* = 0.88) (Supplementary Figures S2A,B). The forest plot is showed in Figure 3. The stability of the MR estimates was further validated through a leave-out test (Supplementary Figures S3A,B).

### 3.2 MR analysis of pneumoconiosis on RA

**Figure 4:**
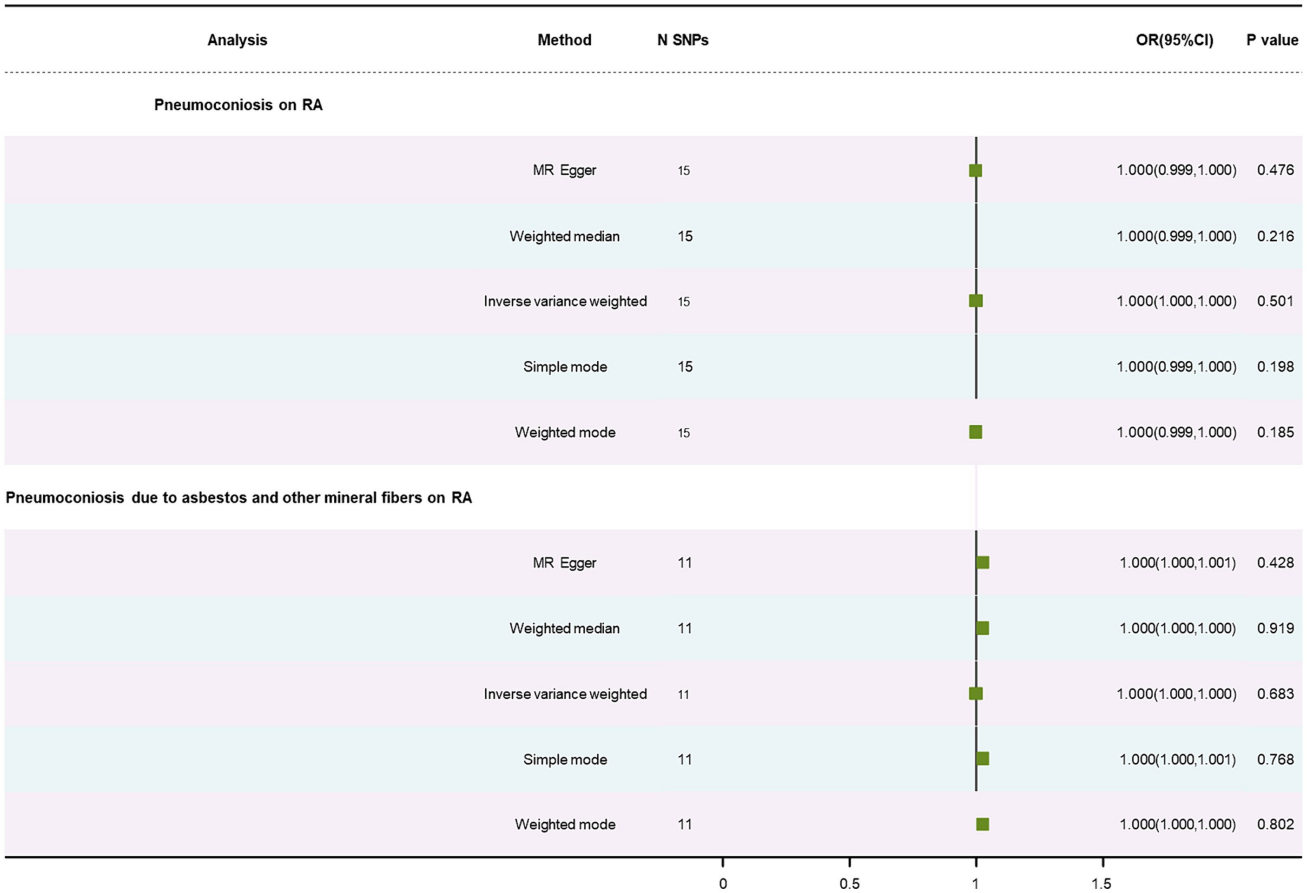
Forest plot of SNPs associated with pneumoconiosis and pneumoconiosis due to asbestos and their risk on RA.

Using the IVW approach, no statistically significant association was found in results on RA, presented in Table 1 (OR: 1.00, *p* = 0.50; OR: 1.00, *p* = 0.68). The WM and the MR-Egger method similarly failed to find this relationship (Supplementary Figures S1C,D). There was no significant heterogeneity (*p* = 0.38; *p* = 0.18) and no horizontal pleiotropy (*p* = 0.66; *p* = 0.19) (Supplementary Figures S2C,D). The forest plot is depicted in Figure 4. A leave-out test was used to confirm the stability of the MR estimations (Supplementary Figures S3C,D).

### 3.3 MR analysis of pneumoconiosis on gout

**Figure 5:**
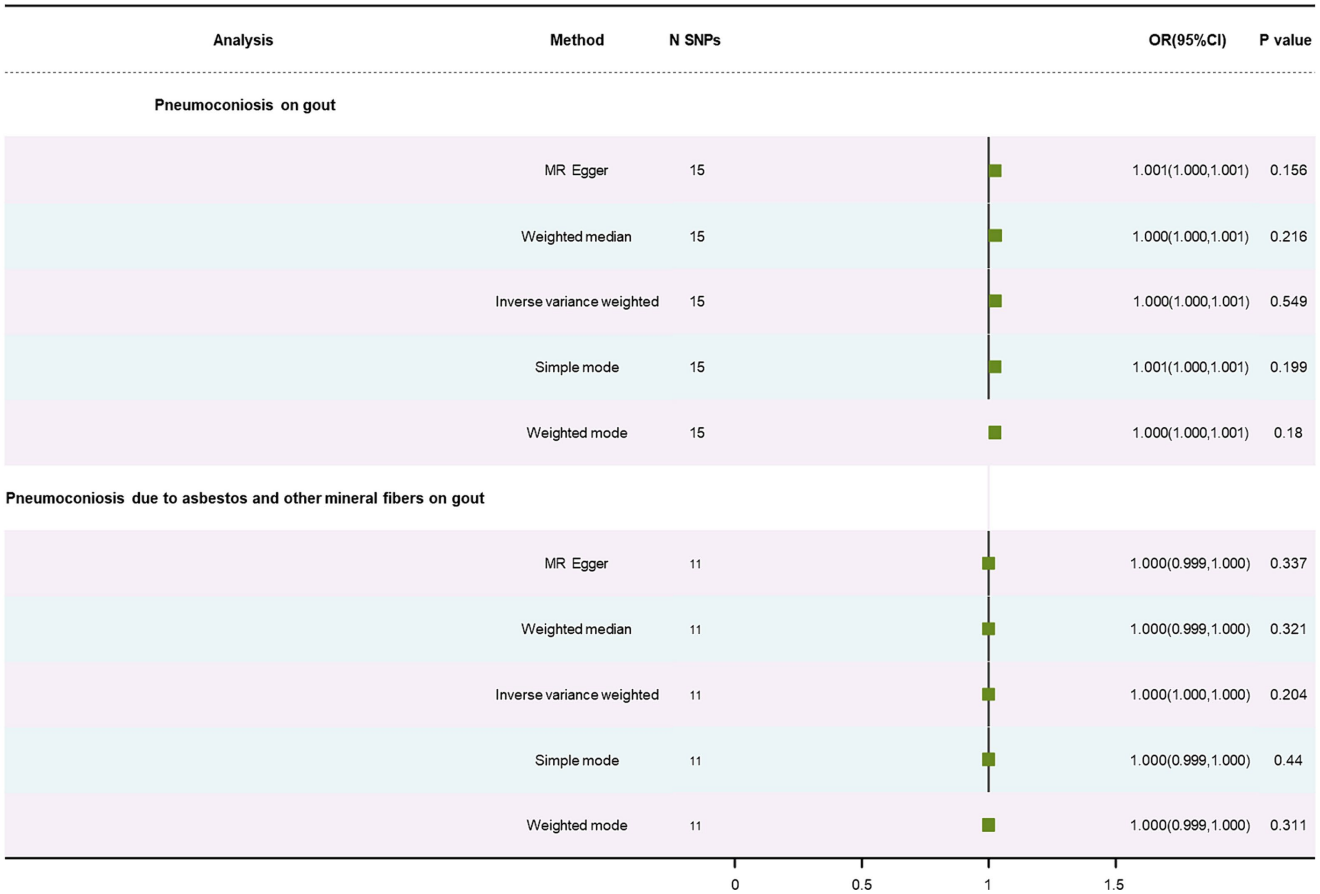
Forest plot of SNPs associated with pneumoconiosis and pneumoconiosis due to asbestos and their risk on gout.

No causal relationship was found in results on gout, displayed in Table 1, using the IVW method (OR: 1.00, *p* = 0.55; OR: 1.00, *p* = 0.20). This correlation was also failed to find using the WM method and MR-Egger approach (Supplementary Figures S1E,F). Pneumoconiosis had heterogeneous outcomes for gout, while pneumoconiosis due to asbestos and other mineral fibers did not (*p* = 0.01; *p* = 0.47). Our analysis indicated no horizontal pleiotropy (*p* = 0.19; *p* = 0.77) (Supplementary Figures S2E,F). The forest plot is showed in Figure 5. A leave-out test was applied to assess the stability of the MR estimates (Supplementary Figures S3E,F).

## 4 Discussion

To our knowledge, this is the first study to investigate the causal connection between pneumoconiosis and inflammatory immune diseases. Our results indicates that pneumoconiosis and SLE, RA, and gout are not causally related. Nevertheless, further research is needed to validate and clarify the mechanism.

Although the etiology and pathogenesis of inflammatory immune diseases are still unclear, they might be related to environmental factors. In some investigations, the development of RA, SLE, and other inflammatory immunological disorders is linked to occupational exposure to external airborne agents (EAA) (22). Workers in different manufacturing and construction sectors, as well as miners and quarry workers, might be frequently exposed to EAA, including cSiO_2_, asbestos, and steam (23). Pneumoconiosis is triggered directly by exposure to cSiO2 (a major factor), asbestos, etc. These elements have been linked to an elevated incidence and severity of inflammatory immunological disorders (2, 24). These environmental pollutants can be used as a molecular pattern that causes damage once they enter the human body, producing the autoinflammatory response that is typified by increased production of reactive oxygen species, increased macrophage flux, NF-κB activation, tumor necrosis factor, IL-1β, and IL-6, among other pro-inflammatory cytokines (25). These triggers cause B cells and dendritic cells to get stimulated, which in turn produces a lot of antibodies and autoreactive T lymphocytes. This destroys immunological tolerance and causes inflammatory immune diseases (26, 27).

New Zealand mixed mice exposed to silicate showed reduced blood IgG, a biomarker of systemic inflammation, and lifted growth of TNF-α, IL-6, B-cell activating factor, and IgM. Further still, cSiO_2_ also causes an autoantibody reaction to autoantigens linked to RA and SLE (28, 29). Pneumoconiosis patients’ serum cytokine analysis also led to some discoveries. Serum IL-6 appeared to be included in the same factor as ANCA levels and antinuclear antibody titers. Apart from boosting responder T cells and lowering Treg cells, IL-6 also contributes a great deal to T helper cell polarization towards Th17 cells, preventing cSiO_2_-induced lung inflammation via an IL-1B-dependent pathway (30, 31). The predominant factor influencing the level of inflammation is the fraction of quartz, a part of the respirable dust responsible for coal dust (32). However, a number of studies consistently demonstrate that the development of pneumoconiosis is not influenced by the quartz concentration of mixed dust. For instance, pneumoconiosis is less common in German coal mines where quartz concentrations are greater (33). Conversely, a coal mine with a lower proportion of quartz was shown to have a higher prevalence of pneumoconiosis (34). In French miners, high quartz concentrations have not been linked to pulmonary fibrosis (35). A review goes into great length on animal studies, *in vitro* evaluations, and epidemiological evidence supporting the hypothesis that iron concentration in coal, not quartz, raises the risk of pneumoconiosis in coal miners (36). It also mentioned that a potential biomarker might be the changed ferritin levels that coal workers experience after taking in coal dust. Moreover, clay samples significantly inhibited quartz-induced lung inflammation (37). Thus, the entire burden of pneumoconiosis may be entirely attributed to occupational exposure to gases, smoke, and particulate matter through risk factors (38). Rather than inflammation, the degree of occupational exposure concentration and exposure duration plays an essential part in the development and course of pneumoconiosis (39). Information on blood markers of systemic inflammation is not available in the database. Lung inflammation due to quartz content in inhaled dust may not cause a high lung burden or develop pneumoconiosis (40). Therefore, our study suggests that there is no causal relationship between pneumoconiosis and the three inflammatory immune diseases.

There are several limitations in previous observational studies. Firstly, the number of cases of SLE, RA, and gout caused by pneumoconiosis may have been underestimated and misclassified because the patients included were mainly diagnosed as outpatients. Secondly, many studies lacked case information and were therefore unable to control for relevant confounders, including family history and lifestyles. Thirdly, the history of dust exposure was incomplete, including information regarding the usage of personal protective equipment, which is the optimum approach for reducing cSiO2 exposure and preventing pneumoconiosis in workers.

There are also some limitations in our study to consider. First, we relaxed the selection criterion (*p* < 5 × 10^−6^, *r*^2^ = 0.001, kb = 10,000) as there were not numerous SNPS that fit the inclusion requirements. This might result in some false positives. Besides, the study focused on European populations, and it is unknown if other populations show the same link. Finally, we did not obtain GWAS data for pneumoconiosis caused by other exposures, and etiological categorization could not be analyzed. In conclusion, we did not find a causal relationship between pneumoconiosis and the three inflammatory immune diseases.

## Data availability statement

The original contributions presented in the study are included in the article/Supplementary material, further inquiries can be directed to the corresponding author.

## Author contributions

Y-JD: Writing – original draft. Z-WL: Writing – original draft. K-DL: Writing – original draft. Y-YuW: Writing – original draft. HW: Writing – original draft. R-GH: Writing – original draft. XJ: Writing – original draft. Y-YuaW: Writing – original draft. JW: Writing – original draft. A-YG: Writing – original draft. B-ZL: Writing – review & editing.

## Footnotes

^1^ https://gwas.mrcieu.ac.uk/

^2^ https://www.finngen.fi/fi

^3^ https://www.ebi.ac.uk/
