# Supplementary material for: No causal association between pneumoconiosis and three inflammatory immune diseases: a Mendelian randomization study
Source: Front Public Health. 2024 Mar 27;12:1373044. doi: 10.3389/fpubh.2024.1373044 (PMC11004292; doi:10.3389/fpubh.2024.1373044)
Supplement: Supplementary file 1 [file Data_Sheet_1.docx]

***Supplementary Material***

**No causal association between** **pneumoconiosis and three inflammatory immune diseases: a Mendelian randomization study**

**Yu-Jie Du^1,2†^, Zhang-Wei Lu^1, 2†^, Kai-Di Li^1,2^, Yi-Yu Wang^1,2^, Hong Wu^1,2^, Xue Jin^1,2^, Rong-Gui Huang^1,2^, Yi-Yuan Wang^1,2^, Jing Wang^1,2^, An-Yi Geng^1,2^, and Bao-Zhu Li^1,2,3*^**

*** Correspondence:**

Bao-Zhu Li

[lbz88730@163.com](mailto:lbz88730@163.com).


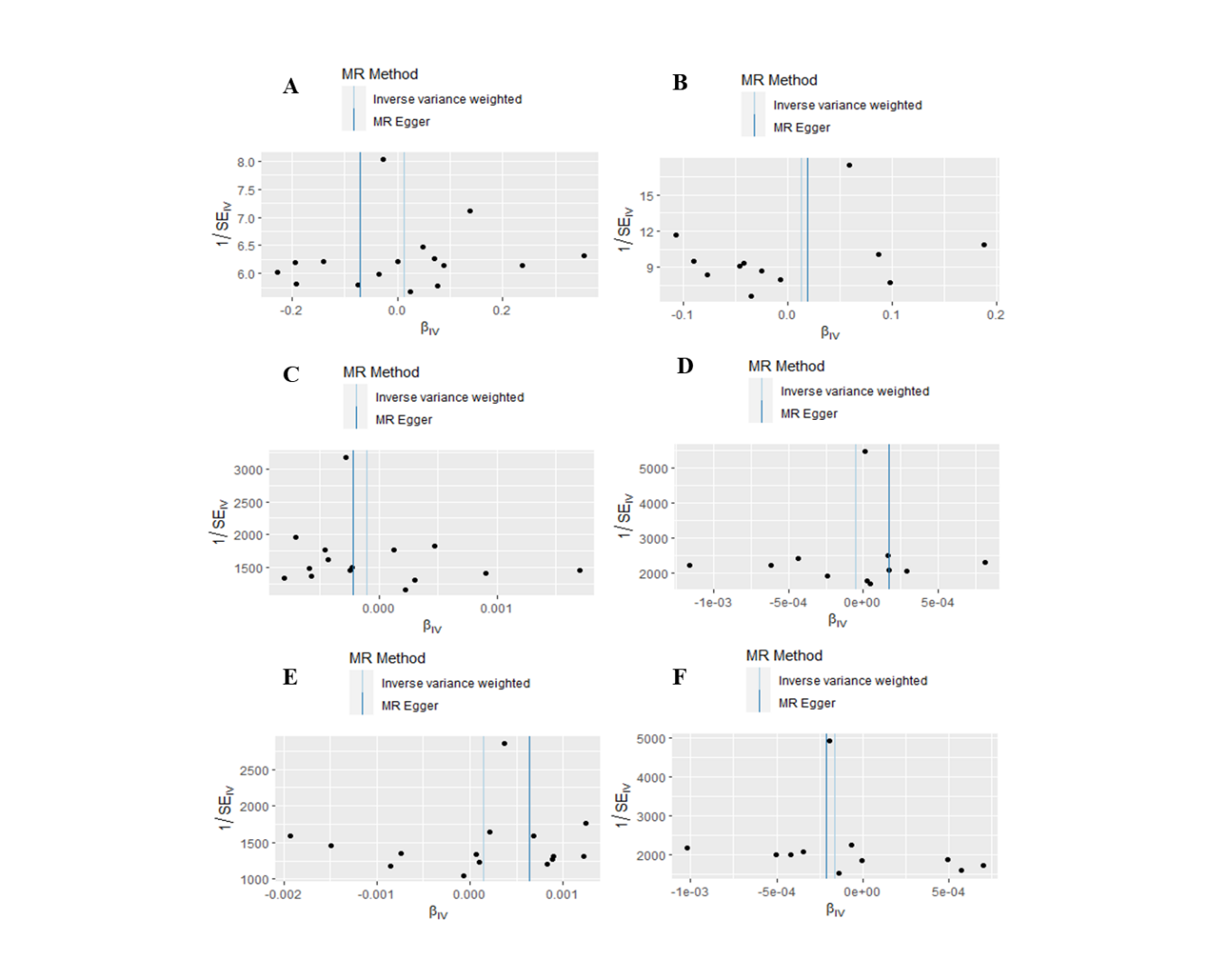


**Supplementary Figure S1** Scatter plots for pneumoconiosis and pneumoconiosis due to asbestos and other mineral fibers on SLE, RA and gout

1. Scatter plot for pneumoconiosis on SLE
2. Scatter plot for pneumoconiosis due to asbestos and other mineral fibers on SLE
3. Scatter plot for pneumoconiosis on RA
4. Scatter plot for pneumoconiosis due to asbestos and other mineral fibers on RA
5. Scatter plot for pneumoconiosis on gout
6. Scatter plot for pneumoconiosis due to asbestos and other mineral fibers on gout

MR, Mendelian randomization; SE, standard error.

**
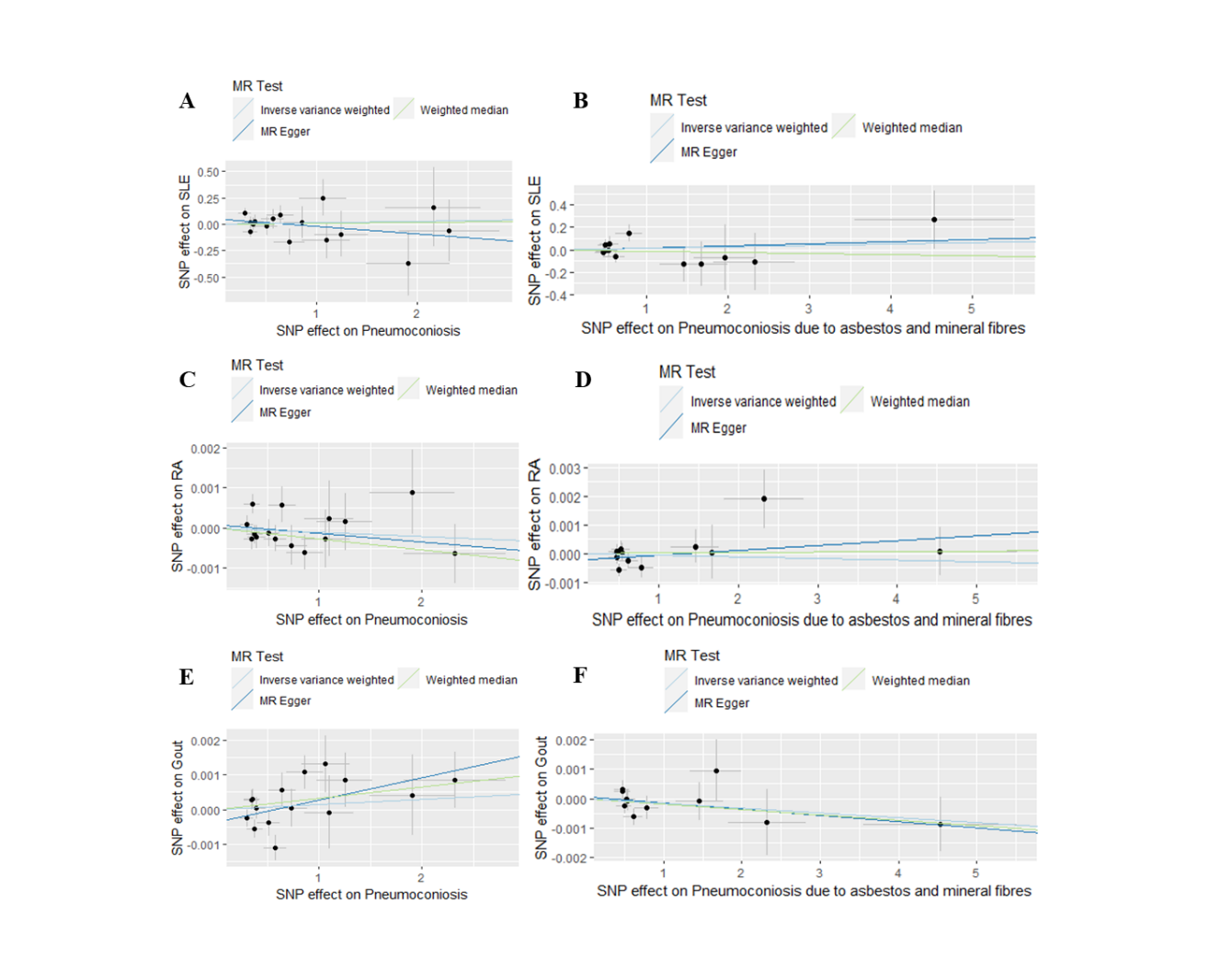
**

**Supplementary Figure S2**

Funnel plots for pneumoconiosis and pneumoconiosis due to asbestos and other mineral fibers on SLE, RA and gout

MR, Mendelian randomization; SE, standard error.

(A) Funnel plot for pneumoconiosis on SLE

(B) Funnel plot for pneumoconiosis due to asbestos and other mineral fibers on SLE

(C) Funnel plot for pneumoconiosis on RA

(D) Funnel plot for pneumoconiosis due to asbestos and other mineral fibers on RA

(E) Funnel plot for pneumoconiosis on gout

(F) Funnel plot for pneumoconiosis due to asbestos and other mineral fibers on gout

MR, Mendelian randomization; SE, standard error.


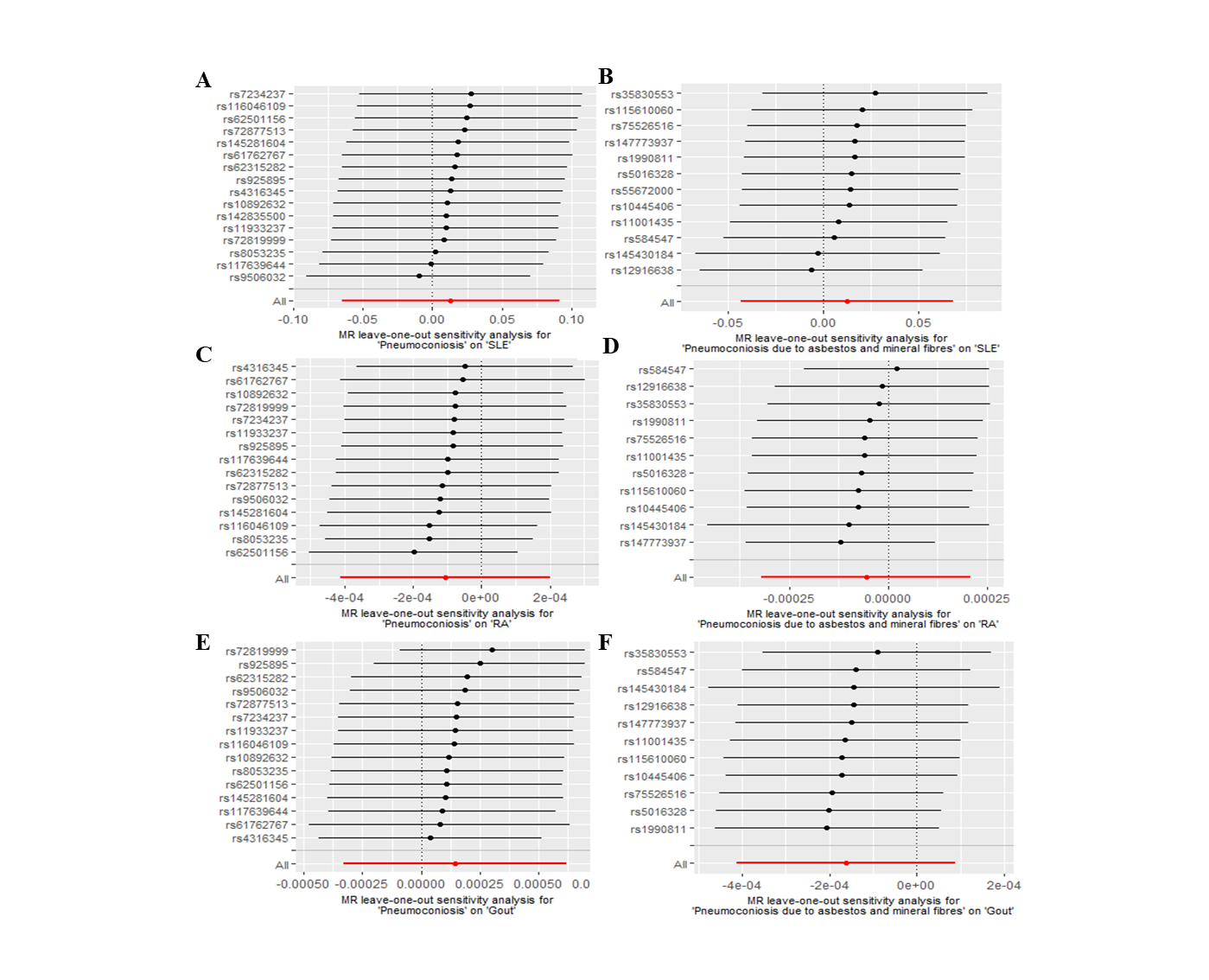


**Supplementary Figure S3** Leave‐one‐out sensitivity analysis for pneumoconiosis and pneumoconiosis due to asbestos and other mineral fibers on SLE, RA and gout

MR: Mendelian randomization; SNP: single‐nucleotide polymorphism.

**(A)** Leave‐one‐out sensitivity analysis for pneumoconiosis on SLE

**(B)** Leave‐one‐out sensitivity analysis due to asbestos and other mineral fibers on SLE

**(C)** Leave‐one‐out sensitivity analysis for pneumoconiosis on RA

**(D)** Leave‐one‐out sensitivity analysis for pneumoconiosis due to asbestos and other mineral fibers on RA

**(E)** Leave‐one‐out sensitivity analysis for pneumoconiosis on gout

**(F)** Leave‐one‐out sensitivity analysis for pneumoconiosis due to asbestos and other mineral fibers on gout

MR, Mendelian randomization; SE, standard error.

**Supplementary Table S1** Characteristics of the genetic instrumental variables used in the two sample MR analysis of pneumoconiosis and pneumoconiosis due to asbestos and other mineral fibers on SLE

| Exposure | SNP | A1 | A2 | β | SE | *P* | N | F | R^2^ |
| --- | --- | --- | --- | --- | --- | --- | --- | --- | --- |
| E1 | rs10445406 | A | G | 0.53 | 0.11 | 3.93E-06 | 217101 | 23872 | 0.10 |
| E1 | rs11001435 | C | T | 0.54 | 0.12 | 2.94E-06 | 217101 | 24857 | 0.10 |
| E1 | rs115610060 | G | T | 1.46 | 0.30 | 1.12E-06 | 217101 | 32667 | 0.13 |
| E1 | rs12916638 | T | C | 0.78 | 0.16 | 8.88E-07 | 217101 | 29244 | 0.12 |
| E1 | rs145430184 | G | C | 4.53 | 0.98 | 3.73E-06 | 217101 | 49666 | 0.19 |
| E1 | rs147773937 | A | G | 2.32 | 0.50 | 3.28E-06 | 217101 | 33899 | 0.14 |
| E1 | rs1990811 | A | C | -0.47 | 0.10 | 3.81E-06 | 217101 | 22639 | 0.09 |
| E1 | rs35830553 | C | G | 0.61 | 0.12 | 8.34E-07 | 217101 | 28283 | 0.12 |
| E1 | rs5016328 | C | G | -0.47 | 0.10 | 2.15E-06 | 217101 | 24277 | 0.10 |
| E1 | rs55672000 | A | G | 1.96 | 0.38 | 3.37E-07 | 217101 | 42964 | 0.17 |
| E1 | rs584547 | A | G | 0.50 | 0.10 | 2.74E-07 | 217101 | 29237 | 0.12 |
| E1 | rs75526516 | A | G | 1.67 | 0.33 | 5.96E-07 | 217101 | 39908 | 0.16 |
| E1 | rs10445406 | A | G | 0.53 | 0.11 | 3.93E-06 | 217101 | 23872 | 0.10 |
| E2 | rs10892632 | G | T | 0.34 | 0.07 | 2.54E-06 | 479040 | 24644 | 0.05 |
| E2 | rs116046109 | A | G | 1.91 | 0.41 | 3.63E-06 | 479040 | 34973 | 0.07 |
| E2 | rs117639644 | A | T | 1.06 | 0.23 | 3.97E-06 | 479040 | 29213 | 0.06 |
| E2 | rs11933237 | T | C | 0.39 | 0.09 | 4.93E-06 | 479040 | 21148 | 0.04 |
| E2 | rs142835500 | T | C | 2.16 | 0.47 | 4.38E-06 | 479040 | 34058 | 0.07 |
| E2 | rs145281604 | A | G | 1.25 | 0.27 | 3.38E-06 | 479040 | 32702 | 0.06 |
| E2 | rs4316345 | A | T | 0.86 | 0.18 | 1.39E-06 | 479040 | 31515 | 0.06 |
| E2 | rs61762767 | A | G | 2.32 | 0.50 | 3.19E-06 | 479040 | 147235 | 0.24 |
| E2 | rs62315282 | A | C | 0.51 | 0.11 | 1.89E-06 | 479040 | 27833 | 0.05 |
| E2 | rs62501156 | C | G | 0.35 | 0.07 | 3.26E-06 | 479040 | 210978 | 0.04 |
| E2 | rs7234237 | G | T | 0.73 | 0.15 | 1.96E-06 | 479040 | 28561 | 0.06 |
| E2 | rs72819999 | T | A | 0.57 | 0.11 | 3.68E-07 | 479040 | 34563 | 0.07 |
| E2 | rs72877513 | G | C | 1.10 | 0.24 | 4.13E-06 | 479040 | 20344 | 0.04 |
| E2 | rs8053235 | T | C | 0.64 | 0.13 | 1.78E-06 | 479040 | 26290 | 0.05 |
| E2 | rs925895 | T | C | 0.37 | 0.08 | 2.80E-06 | 479040 | 30180 | 0.06 |
| E2 | rs9506032 | C | A | -0.30 | 0.06 | 4.67E-06 | 479040 | 21367 | 0.04 |

A1: effect_allele; A2: other_allele; *β*: beta; F: F‐statistics; N: sample size; SE: standard error; SNP: single‐nucleotide polymorphism; E1: pneumoconiosis due to asbestos and other mineral fibers; E2: pneumoconiosis.

**Supplementary Table S2** Characteristics of the genetic instrumental variables used in the two sample MR analysis of pneumoconiosis and pneumoconiosis due to asbestos and other mineral fibers on RA

| Exposure | SNP | A1 | A2 | β | SE | *P* | N | F | R^2^ |
| --- | --- | --- | --- | --- | --- | --- | --- | --- | --- |
| E1 | rs10445406 | A | G | 0.53 | 0.11 | 3.93E-06 | 217101 | 23872 | 0.10 |
| E1 | rs11001435 | C | T | 0.54 | 0.12 | 2.94E-06 | 217101 | 24857 | 0.10 |
| E1 | rs115610060 | G | T | 1.46 | 0.30 | 1.12E-06 | 217101 | 32667 | 0.13 |
| E1 | rs12916638 | T | C | 0.78 | 0.16 | 8.88E-07 | 217101 | 29244 | 0.12 |
| E1 | rs145430184 | G | C | 4.53 | 0.98 | 3.73E-06 | 217101 | 49666 | 0.19 |
| E1 | rs147773937 | A | G | 2.32 | 0.50 | 3.28E-06 | 217101 | 33899 | 0.14 |
| E1 | rs1990811 | A | C | -0.47 | 0.10 | 3.81E-06 | 217101 | 22639 | 0.09 |
| E1 | rs35830553 | C | G | 0.61 | 0.12 | 8.34E-07 | 217101 | 28283 | 0.12 |
| E1 | rs5016328 | C | G | -0.47 | 0.10 | 2.15E-06 | 217101 | 24277 | 0.10 |
| E1 | rs584547 | A | G | 0.50 | 0.10 | 2.74E-07 | 217101 | 29237 | 0.12 |
| E1 | rs75526516 | A | G | 1.67 | 0.33 | 5.96E-07 | 217101 | 39908 | 0.16 |
| E2 | rs10892632 | G | T | 0.34 | 0.07 | 2.54E-06 | 479040 | 24644 | 0.05 |
| E2 | rs116046109 | A | G | 1.91 | 0.41 | 3.63E-06 | 479040 | 34973 | 0.07 |
| E2 | rs117639644 | A | T | 1.06 | 0.23 | 3.97E-06 | 479040 | 29213 | 0.06 |
| E2 | rs11933237 | T | C | 0.39 | 0.09 | 4.93E-06 | 479040 | 21148 | 0.04 |
| E2 | rs145281604 | A | G | 1.25 | 0.27 | 3.38E-06 | 479040 | 32702 | 0.06 |
| E2 | rs4316345 | A | T | 0.86 | 0.18 | 1.39E-06 | 479040 | 31515 | 0.06 |
| E2 | rs61762767 | A | G | 2.32 | 0.50 | 3.19E-06 | 479040 | 147235 | 0.24 |
| E2 | rs62315282 | A | C | 0.51 | 0.11 | 1.89E-06 | 479040 | 27833 | 0.05 |
| E2 | rs62501156 | C | G | 0.35 | 0.07 | 3.26E-06 | 479040 | 21098 | 0.04 |
| E2 | rs7234237 | G | T | 0.73 | 0.15 | 1.96E-06 | 479040 | 28561 | 0.06 |
| E2 | rs72819999 | T | A | 0.57 | 0.11 | 3.68E-07 | 479040 | 34563 | 0.07 |
| E2 | rs72877513 | G | C | 1.10 | 0.24 | 4.13E-06 | 479040 | 20344 | 0.04 |
| E2 | rs8053235 | T | C | 0.64 | 0.13 | 1.78E-06 | 479040 | 26290 | 0.05 |
| E2 | rs925895 | T | C | 0.37 | 0.08 | 2.80E-06 | 479040 | 30180 | 0.06 |
| E2 | rs9506032 | C | A | -0.30 | 0.06 | 4.67E-06 | 479040 | 21367 | 0.04 |

A1: effect_allele; A2: other_allele; *β*: beta; F: F‐statistics; N: sample size; SE: standard error; SNP: single‐nucleotide polymorphism; E1: pneumoconiosis due to asbestos and other mineral fibers; E2: pneumoconiosis.

**Supplementary Table S3** Characteristics of the genetic instrumental variables used in the two sample MR analysis of pneumoconiosis and pneumoconiosis due to asbestos and other mineral fibers on gout

| Exposure | SNP | A1 | A2 | β | SE | P | N | F | R^2^ |
| --- | --- | --- | --- | --- | --- | --- | --- | --- | --- |
| E1 | rs10445406 | A | G | 0.53 | 0.11 | 3.93E-06 | 217101 | 23872.14 | 0.10 |
| E1 | rs11001435 | C | T | 0.54 | 0.12 | 2.94E-06 | 217101 | 24857.11 | 0.10 |
| E1 | rs115610060 | G | T | 1.46 | 0.30 | 1.12E-06 | 217101 | 32666.59 | 0.13 |
| E1 | rs12916638 | T | C | 0.78 | 0.16 | 8.88E-07 | 217101 | 29243.63 | 0.12 |
| E1 | rs145430184 | G | C | 4.53 | 0.98 | 3.73E-06 | 217101 | 49665.68 | 0.19 |
| E1 | rs147773937 | A | G | 2.32 | 0.50 | 3.28E-06 | 217101 | 33899.30 | 0.14 |
| E1 | rs1990811 | A | C | -0.47 | 0.10 | 3.81E-06 | 217101 | 22638.65 | 0.09 |
| E1 | rs35830553 | C | G | 0.61 | 0.12 | 8.34E-07 | 217101 | 28282.67 | 0.12 |
| E1 | rs5016328 | C | G | -0.47 | 0.10 | 2.15E-06 | 217101 | 24276.63 | 0.10 |
| E1 | rs584547 | A | G | 0.50 | 0.10 | 2.74E-07 | 217101 | 29236.81 | 0.12 |
| E1 | rs75526516 | A | G | 1.67 | 0.33 | 5.96E-07 | 217101 | 39907.78 | 0.16 |
| E2 | rs10892632 | G | T | 0.34 | 0.07 | 2.54E-06 | 479040 | 24644 | 0.05 |
| E2 | rs116046109 | A | G | 1.91 | 0.41 | 3.63E-06 | 479040 | 34973 | 0.07 |
| E2 | rs117639644 | A | T | 1.06 | 0.23 | 3.97E-06 | 479040 | 29213 | 0.06 |
| E2 | rs11933237 | T | C | 0.39 | 0.09 | 4.93E-06 | 479040 | 21148 | 0.04 |
| E2 | rs145281604 | A | G | 1.25 | 0.27 | 3.38E-06 | 479040 | 32702 | 0.06 |
| E2 | rs4316345 | A | T | 0.86 | 0.18 | 1.39E-06 | 479040 | 31515 | 0.06 |
| E2 | rs61762767 | A | G | 2.32 | 0.50 | 3.19E-06 | 479040 | 147235 | 0.24 |
| E2 | rs62315282 | A | C | 0.51 | 0.11 | 1.89E-06 | 479040 | 27833 | 0.05 |
| E2 | rs62501156 | C | G | 0.35 | 0.07 | 3.26E-06 | 479040 | 21098 | 0.04 |
| E2 | rs7234237 | G | T | 0.73 | 0.15 | 1.96E-06 | 479040 | 28561 | 0.06 |
| E2 | rs72819999 | T | A | 0.57 | 0.11 | 3.68E-07 | 479040 | 34563 | 0.07 |
| E2 | rs72877513 | G | C | 1.10 | 0.24 | 4.13E-06 | 479040 | 20344 | 0.04 |
| E2 | rs8053235 | T | C | 0.64 | 0.13 | 1.78E-06 | 479040 | 26290 | 0.05 |
| E2 | rs925895 | T | C | 0.37 | 0.08 | 2.80E-06 | 479040 | 30180 | 0.06 |
| E2 | rs9506032 | C | A | -0.30 | 0.06 | 4.67E-06 | 479040 | 21367 | 0.04 |

A1: effect_allele; A2: other_allele; *β*: beta; F: F‐statistics; N: sample size; SE: standard error; SNP: single‐nucleotide polymorphism; E1: pneumoconiosis due to asbestos and other mineral fibers; E2: pneumoconiosis.
